# Editorial: Digital transformation in sports coaching—enhancing coach learning and athlete development

**DOI:** 10.3389/fspor.2026.1785591

**Published:** 2026-03-05

**Authors:** Koon Teck Koh, Tarkington J. Newman, Fernando Santos

**Affiliations:** 1 National Institute of Education, Nanyang Technological University, Singapore, Singapore; 2 Sport Social Work Research Lab, College of Social Work, University of Kentucky, Lexington, KY, United States; 3 Centro de Investigação e Inovação em Educação, inED, Escola Superior de Educação, Instituto Politécnico do Porto, Porto, Portugal

**Keywords:** artificial intelligence, coach learning, coaching knowledge, pedagogy, technology

Digital technologies are increasingly reshaping the landscape of sports coaching, influencing how coaches learn, communicate, make decisions, and support athlete development. Although technological innovation in sports has been widely examined in relation to athlete monitoring, performance optimization, and data analytics, less attention has focused on understanding how digital innovation shapes coach learning, professional judgment, and pedagogical practice. Moreover, while digital technologies have the potential to expand access, connectivity, and flexibility, persistent challenges remain related to contextual relevance, pedagogical coherence, and professional identity. These gaps limit the capacity to conceptualize technology as a meaningful, theoretically-grounded, and ethically-informed component of coach development. In response, this Research Topic seeks to advance empirical and theoretical understandings of how digital technologies can support coach learning, enhance coaching practice, and contribute to holistic athlete development in contemporary society. This editorial synthesizes contributions across three interconnected areas: digital transformation in sports coaching, enhancing coach learning through technology, and enhancing athlete development in contemporary society.

## Digital transformation in sports coaching

Digital transformation in sports coaching extends beyond the introduction of new strategies or tools and represents a fundamental shift in how knowledge is produced and accessed. Technologies such as online learning environments, video-based feedback systems, wearable devices, performance dashboards, and artificial intelligence (AI) tools are increasingly embedded across participation, developmental, and elite sport coaching systems. Several contributions within this Research Topic demonstrate how technologies can expand opportunities for learning and practice, particularly in contexts where traditional provision is constrained. LeCrom and Smith (1) examine virtual coach education in rural and remote communities and demonstrate that online platforms can mitigate geographic isolation and resource limitations. However, findings also highlight that structural inequities (e.g., differences in structure, institutional support, and digital literacy) cannot be resolved through technology alone. Indeed, technology can enable and constrain practice simultaneously. Digital tools shape what knowledge is prioritized, how learning is structured, and whose expertise is legitimized. Accordingly, digital transformation in sports coaching must be understood as an ethical endeavor, rather than a purely pedagogical and/or technological venture. This positioning advances a more critical and sustainable approach to digital transformation in coaching.

## Enhancing coach learning through technology

A central concern in contemporary coaching research is understanding the processes and mechanisms through which coaches learn. Scholars recognize that coach learning extends beyond formal education programmes and occurs across formal, non-formal, and informal settings, including everyday practices and peer interactions. Increasingly, digital communities and other coaching forums are being leveraged (2). Concurrently, scholarship also highlights the conceptual ambiguity surrounding professional competence, underscoring the need for clearer theoretical positioning when designing learning frameworks (3). Several contributions within this Research Topic propose novel approaches to enhance coach learning. O'Brien and Prentice (4) examine AI-based generative tools that challenge traditional assumptions about authoritative knowledge sources and invite reflection on how coach learning is increasingly distributed across human and non-human actors. Bates et al. (5) investigate the impact of coach mental health training that emphasizes well-being as a core component of coaching competence and professional responsibility. Collectively, these contributions invite scholars and practitioners to rethink not only how coaches learn, but what learning is required for the future of coaching.

## Enhancing athlete development in contemporary society

Digital transformation also has significant implications for how athlete development is conceptualized and actualized. Traditional performance-oriented models are increasingly complemented by holistic approaches that emphasize psychological well-being, social development, educational engagement, and long-term participation (6). Digital tools provide new mechanisms to support these broader developmental aims. Hagiwara et al. validate a web-based version of the Dual Career Competency Questionnaire for Athletes, demonstrating how digital assessment tools can inform coach-led support for balancing academic and sporting demands. Richards et al. (7) illustrate how video-based analysis integrated with cognitive task analysis can enhance shared understanding and decision-making within elite teams. Koh et al. (8) further demonstrate how mobile applications can support reflective practice and self-directed learning for coaches, indirectly enhancing athlete development through improved coaching quality. Collectively, these studies highlight that although digital technologies can augment coaching practice and athlete development, such innovations cannot replace the relational, ethical, and contextual dimensions that underpin effective coaching.

## Conclusion and future directions

This Research Topic demonstrates that digital transformation in sports coaching is a complex, multi-layered process reshaping coach learning and athlete development. Across the individual contributions, four themes emerged: equity and access; coach agency and professional judgment; blended and contextualized learning; and human–AI collaboration. Addressing these areas in the future will be essential to ensure that digital innovation enhances sports coaching in ethical, inclusive, and developmentally meaningful ways (4).
